# Rare copy number variations affecting the synaptic gene *DMXL2* in neurodevelopmental disorders

**DOI:** 10.1186/s11689-019-9263-3

**Published:** 2019-02-07

**Authors:** Gregory Costain, Susan Walker, Bob Argiropoulos, Danielle A. Baribeau, Anne S. Bassett, Erik Boot, Koen Devriendt, Barbara Kellam, Christian R. Marshall, Aparna Prasad, Moises A. Serrano, D. James Stavropoulos, Hope Twede, Joris R. Vermeesch, Jacob A. S. Vorstman, Stephen W. Scherer

**Affiliations:** 1 Division of Clinical and Metabolic Genetics, The Hospital for Sick Children, Toronto, ON Canada; 2 Medical Genetics Residency Training Program, University of Toronto, Toronto, ON Canada; 3 The Centre for Applied Genomics, The Hospital for Sick Children, Toronto, ON Canada; 4 Program in Genetics and Genome Biology, The Hospital for Sick Children, Toronto, ON Canada; 5 Department of Medical Genetics, University of Calgary Cumming School of Medicine, Calgary, AB Canada; 6 Department of Psychiatry, University of Toronto, Toronto, ON Canada; 7 The Dalglish Family 22q Clinic, Toronto General Hospital, Toronto, ON Canada; 8 Department of Human Genetics, KU Leuven, Leuven, Flanders Belgium; 9 Genome Diagnostics, Department of Paediatric Laboratory Medicine, The Hospital for Sick Children, Toronto, ON Canada; 10 Lineagen, Inc, 2677 East Parleys Way, Salt Lake City, UT 84109 USA; 11 Autism Research Unit, The Hospital for Sick Children, Toronto, ON Canada; 12 Department of Molecular Genetics and McLaughlin Centre, University of Toronto, Toronto, ON Canada

**Keywords:** Copy number variation, Genome sequencing, Autism, ADHD, *DMXL2*, *GRIK5*

## Abstract

**Background:**

Ultra-rare genetic variants, including non-recurrent copy number variations (CNVs) affecting important dosage-sensitive genes, are important contributors to the etiology of neurodevelopmental disorders (NDDs). Pairing family-based whole-genome sequencing (WGS) with detailed phenotype data can enable novel gene associations in NDDs.

**Methods:**

We performed WGS of six members from a three-generation family, where three individuals each had a spectrum of features suggestive of a NDD. CNVs and sequence-level variants were identified and further investigated in disease and control databases.

**Results:**

We identified a novel 252-kb deletion at 15q21 that overlaps the synaptic gene *DMXL2* and the gene *GLDN*. The microdeletion segregated in NDD-affected individuals. Additional rare inherited and de novo sequence-level variants were found that may also be involved, including a missense change in *GRIK5*. Multiple CNVs and loss-of-function sequence variants affecting *DMXL2* were discovered in additional unrelated individuals with a range of NDDs.

**Conclusions:**

Disruption of *DMXL2* may predispose to NDDs including autism spectrum disorder. The robust interpretation of private variants requires a multifaceted approach that incorporates multigenerational pedigrees and genome-wide and population-scale data.

## Background

Rare genetic variants can contribute to the etiology of common neurodevelopmental disorders (NDDs) such as autism spectrum disorder (ASD) [1–7], attention-deficit/hyperactivity disorder (ADHD) [8, 9], intellectual disability (ID) [10–12], and schizophrenia [13–17]. Marked genetic heterogeneity contributed to the recommendation that genome-wide chromosomal microarray (CMA) be a first-tier genetic test for individuals with selected NDDs [10, 14]. CMA along with whole-genome sequencing (WGS) technology have allowed increasing detection of genomic alterations such as copy number variations (CNVs) affecting important developmental genes [10, 18–20]. These variants are difficult to adjudicate through parental testing alone, as even established “genomic disorders” can demonstrate highly variable neurodevelopmental and neuropsychiatric expression [21, 22]. Many are inherited from a putatively unaffected parent, who may have sub-clinical traits or a history of symptoms suggestive of an undiagnosed psychiatric condition [23]. WGS represents a comprehensive platform for detection of coding and non-coding sequence-level, CNV, structural, and mitochondrial variation [1, 24–26].

There are two main contemporary strategies for discovering NDD genes based on CNVs [18, 27]. One begins with a unique variant in a proband and involves a traditional family-based study design with deep phenotyping and curation of all genomic variants. The other involves searching for genes or loci that are overrepresented in large datasets of rare genetic variation assembled through clinical testing or research consortia. In this study, we combined these “depth” and “breadth” approaches to characterize a small non-recurrent CNV of uncertain clinical significance. We sequenced the genomes of six individuals from a single multiplex NDD family and propose that a novel 15q21 microdeletion is the likely genetic lesion involved, because of haploinsufficiency of a synaptic scaffolding protein encoded by *DMXL2*.

## Methods

### Family recruitment and phenotyping

The adult proband contacted our research group requesting to participate in our ongoing genetic studies of ASD, after clinical CMA testing had revealed a 15q21 deletion of uncertain significance. History provided by the proband was supplemented by review of all available lifetime medical and psychiatric records. The proband and five family members across three generations provided DNA samples for WGS, and written informed consent was obtained for all participants. After the initial submission of this manuscript, one additional family member provided a saliva sample for targeted genetic testing.

### Whole-genome sequencing

WGS methods are as described in detail elsewhere [1, 24]. In brief, WGS was performed at The Centre for Applied Genomics (Toronto, Canada) using DNA extracted from whole blood. Sequencing was performed with the Illumina HiSeq X system and following Illumina’s recommended protocols. Base calling and data analysis were performed using Illumina HiSeq Analysis Software (HAS) version 2-2.5.55.1311. Reads were mapped to the hg19 reference sequence using Isaac alignment software (Isaac alignment software: SAAC00776.15.01.27), and single nucleotide variants (SNVs) and small indel variants were detected using the Isaac variant caller [Isaac Variant Caller (Starling): 2.1.4.2]. These variants were annotated using a custom pipeline based on ANNOVAR [28]. Rare variants were defined as those with ≤ 1% frequency in large public control databases [29–31]. CNVs were detected using the read-depth methods ERDS [32] and CNVnator [33] (using a window size of 500 bp). High-quality CNVs were defined as those greater than 1 kb and detected by both ERDS and CNVnator with more than 50% reciprocal overlap [34], and rare CNVs as those with ≤ 1% frequency in the Autism Speaks MSSNG dataset (probands and parents) [1]. We also annotated CNVs with respect to overlap with gold standard variants in the Database of Genomic Variants (DGV) [35, 36]. For further targeted review of candidate CNVs, we used a control dataset comprised of 10,851 unrelated subjects, with a majority being of European ancestry, who were genotyped on multiple microarray platforms including the Affymetrix Genome-wide Human SNP Array 6.0, Illumina HumanOmni2.5, and Affymetrix CytoScan HD [37, 38]. All genome coordinates in this manuscript refer to NCBI Build 37 (UCSC hg19). Variants of interest were confirmed by PCR and Sanger sequencing (primers available on request) or by CMA.

### Review of public and private databases

We conducted a comprehensive review of public, and non-public clinical and research laboratory, databases of genomic variation in disease (primarily NDD) to identify additional individuals with *DMXL2*, *GLDN*, and *GRIK5* variants. We focused on smaller (< 1 Mb and < 15 genes) CNVs predicted to disrupt any of these genes (i.e., deletions impacting the coding region of the gene, and intragenic duplications), as well as predicted null SNVs and small indels. The denominator of individuals with data available for review is estimated to have been ~ 100,000. This included > 30,000 postnatal cases from Canadian laboratory clinical CMA databases, > 20,000 schizophrenia cases with CMA data from the Psychiatric Genomics Consortium [17], > 18,000 cases with CMA data in DECIPHER [39], > 23,000 cases from the Lineagen Inc. CMA database (Salt Lake City, Utah, USA), and > 6000 cases with CMA data from our ongoing ASD studies and/or WGS data from MSSNG [1].

## Results

### Family members demonstrated variable features of NDDs

**Fig. 1:**
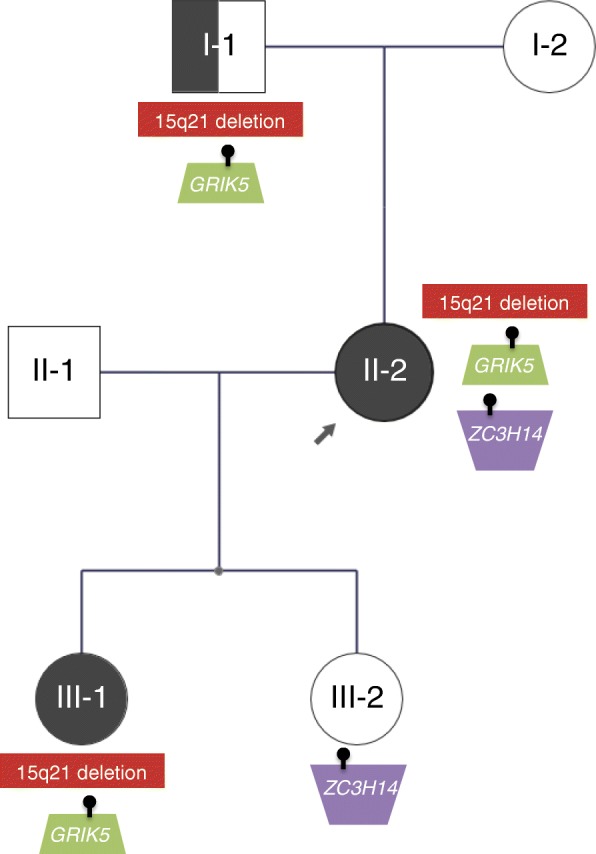
Familial segregation of selected monoallelic rare variants identified by WGS. Individuals II-2 and III-1 have multiple diagnoses including ASD. Individual I-1 was coded as likely affected with a major psychiatric disorder and/or NDD. See text for details. A female paternal first cousin of the proband (not pictured), with possible persistent depressive disorder and no suspected NDD, did not carry the 15q21 deletion or the *GRIK5* missense variant

The proband (II-2; Fig. 1) is a 34-year-old female of Dutch ancestry with a reported history of difficulties with sensory processing and physical contact as a child. During adolescence, symptoms across a broad range of domains were reported, including conduct symptoms, attention deficit, anxiety including panic attacks, and fluctuating mood. She completed preparatory middle-level vocational education (voorbereidend middelbaar beroepsonderwijs or VMBO), the lowest level educational stream in the Netherlands. At age 28 years, Wechsler Adult Intelligence Scale—Third Edition (WAIS-III) showed a marked and unusual [40] difference between verbal IQ (VIQ; 79) and performance IQ (PIQ; 100).

At about age 20, she received pharmacological as well as non-pharmacological treatments for symptoms of anxiety and panic. After the birth of her first child, at age 25, she was treated briefly with an antidepressant for postpartum depression. She was diagnosed with ADHD at age 27 and treated with methylphenidate. At age 33, two independent psychiatric assessments confirmed the previous diagnosis of ADHD. In addition, several aspects of ASD were mentioned and endorsed by questionnaire reports, including reduced empathic abilities, difficulties in social interaction and communication, and difficulties coping with change in her routines. Results of the Social Responsiveness Scale for Adults (reported by self and husband) and the Pervasive Developmental Disorders Screening Questionnaire were in the clinical range. However, these symptoms were evaluated as insufficient to justify a formal diagnosis of ASD. Fluctuations in mood and poor coping skills were interpreted at times as aspects of a borderline personality disorder. A subsequent psychiatric assessment at age 34 years concluded that, in retrospect, ASD was a justified diagnosis based on all available information.

The early developmental trajectory of one of the proband’s daughters (III-1; Fig. 1) was marked by mild cognitive delay and a mixed language disorder (as assessed with the Clinical Evaluation of Language Fundamentals—Fourth Edition). Motor milestones were unremarkable. Her medical history was notable for a febrile seizure at age 3 years and recurrent otitis media, which was associated with temporary hearing loss. She was reported to be a highly anxious infant with a high threshold for pain and hypersensitivity to sensory input. She continued to display moderate behavioral issues as a toddler, both at home and in school, with social and communicative difficulties as well as oppositional, attentional, and anxious features. Early clinical diagnoses included developmental language disorder and disorder of childhood not otherwise specified. At 7 years 11 months, full scale IQ was assessed to be 85, with VIQ 87 and PIQ 86, using the Wechsler Intelligence Scale for Children—Third Edition (WISC-III). The most recent child psychiatric assessment performed at age 9.5 years recorded symptoms including low reciprocity, inadequate social behaviors, and behavioral rituals. Although she scored just below the clinical threshold of the Autism Diagnostic Observation Schedule (ADOS), the overall picture was deemed to justify a clinical diagnosis of ASD, in addition to the language disorder.

**Fig. 2:**
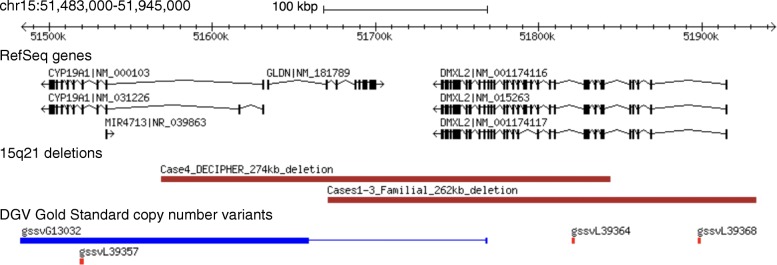
Approximate genomic position of the familial 15q21 deletion and the DECIPHER deletion predicted to disrupt *DMXL2*, visualized using the DGV genome browser [35]. See text and Table 1 for details

The proband’s father (I-1; Fig. 1) was described as having longstanding difficulties with impulsivity/anger, obsessiveness, and other distressing characterological traits that interfered with his functioning. He had similar educational achievement to his daughter and has maintained stable employment without advancement or promotion. He has had at least three “nervous breakdowns” in his life that required him to take a leave of absence from his work. On the basis of this limited history, he was coded prior to WGS as likely affected with a psychiatric disorder and/or NDD. The proband’s other daughter (III-2; Fig. 1) has had normal development, and there are no concerns for ASD. She was diagnosed with Pediatric Acute-onset Neuropsychiatric Syndrome (PANS) after developing motor tics post viral infection. Although the tics initially resolved after a short course of treatment with methylprednisolone, treatment of a recurrence is ongoing. She was coded prior to WGS as unaffected with a NDD. The other two members of the family displayed in Fig. 1 (I-2 and II-1; Fig. 1) are reported to have no major learning difficulties, diagnosed psychiatric conditions, or features of a NDD. There is no known history of unexplained hearing loss in the family [41] nor of infertility or recurrent (> 2) miscarriage, delayed puberty, hypothyroidism, peripheral neuropathy, or short stature [42]. Facial photographs of the proband and her two daughters are available upon request; there are no notable dysmorphic features.

### The familial microdeletion implicates the candidate gene DMXL2

WGS identified the novel 262-kb loss at chromosome 15q21.2 (Fig. 2) in the proband that had been detected on CMA. The deletion was inherited from her father and transmitted to her affected daughter (Fig. 1). This deletion overlaps the entire *DMXL2* gene (GenBank: NM_001174116.1) and the first three exons of *GLDN* (GenBank: NM_181789.3). There were no segmental duplication repeats within 200 kb of the breakpoints. No additional rare CNVs of interest were shared amongst these three family members, or between just the proband and her affected daughter.

*DMXL2* encodes DmX-like protein 2, previously known as Rabconnectin-3. This is a component of the human neocortex post-synaptic density (PSD) proteome [43] that may serve as a scaffold protein on synaptic vesicles, and it is a target of the fragile X mental retardation protein (FMRP) [44, 45]. Multiple lines of evidence, including conditional heterozygous deletion of *Dmxl2* in mouse neurons, suggest that the DMXL2 protein functions in the brain in neuronal and endocrinological homeostasis [42]. The Exome Aggregation Consortium (ExAC) probability of loss-of-function intolerance (pLI) of *DMXL2* is 1.00, with tenfold fewer observed variants than anticipated [31]. *DMXL2* is in a copy number-stable region of the genome; no deletions of *DMXL2* were found in the DGV gold standard variants (Fig. 2) or in our CNV control datasets (see the “Methods” section) [36]. The *DMXL2* gene contains “brain-critical exons” [7]. Two de novo missense variants in *DMXL2* were previously observed in 2517 simplex ASD probands from the Simons Simplex Collection (SSC), with no de novo events in this gene observed in their unaffected siblings [5, 6]. There is also evidence of enrichment for rare sequence variants in *DMXL2* in major depressive disorder [46]. With respect to the other gene overlapped by the 15q21 loss CNV, *GLDN* encodes the protein gliomedin, a secreted cell adhesion molecule involved in the formation of the nodes of Ranvier [47]. In contrast to *DMXL2*, the ExAC pLI is 0.00 and a deletion was observed in one of our control individuals, suggesting there may not be overt clinical consequences for a heterozygous null allele. Biallelic loss of function of *GLDN* causes a lethal congenital contracture syndrome (MIM #617194), and as expected, carrier parents were described as unaffected [48].

### Additional rare sequence variants may shape expression

Considering SNVs and small indels, there were no likely pathogenic or pathogenic variants [49] in established NDD risk genes in any of the six family members. A total of 198 high-quality, rare, predicted damaging variants were shared by the three family members coded as affected (I-1, II-2, III-1; Fig. 1), including 89 that were absent in the proband’s unaffected daughter (III-2). One of these 89 is a missense variant of uncertain significance in *GRIK5* [NM_001301030: c.1840G>A: p.(Ala614Thr)]. It is absent in control databases, affects a highly conserved amino acid, and is predicted to be damaging by in silico programs including SIFT (score 0), PolyPhen-2 (score 0.977), MutationTaster (probability 0.999), and CADD (scaled C-score 32). *GRIK5* encodes an understudied auxiliary subunit of the kainate receptor (glutamate receptor, ionotropic, kainate 5). Dysregulation of other components of this receptor has been associated with psychiatric and neurodevelopmental diseases [50–54]. In ExAC, *GRIK5* is constrained with respect to loss-of-function (pLI = 0.92) and missense (*z* = 3.85) variants. There are no coding CNVs in our control databases impacting *GRIK5*. Similar to *DMXL2*, prior evidence for an association with ASD is derived from the SSC and Autism Sequencing Consortium, where there were three de novo missense variants in the *GRIK5* gene in simplex ASD probands and none in the unaffected SSC siblings [4–6].

We also identified three high confidence de novo exonic variants. In the proband (II-2), there was a 2-bp frameshift deletion in exon 10 of *ZC3H14* [NM_024824.4: c.1339_1340del: p.(Leu447Alafs*8)] and a synonymous substitution in exon 3 of *TRPC4* that was not predicted to affect splicing. *ZC3H14* plays a key role in neurodevelopmental processes [55, 56]. The gene is associated with an autosomal recessive form of ID (MIM #617125) [57], but is also constrained against loss-of-function variants (ExAC pLI = 1), suggesting that it may be sensitive to haploinsufficiency. However, the de novo frameshift variant was transmitted only to the proband’s unaffected daughter (III-2; Fig. 1). The affected daughter (III-1) has a novel de novo missense variant in exon 3 of *DLGAP3* [NM_001080418.2: c.33 T>A: p.(His11Gln)]. This gene is a member of the DLGAP family that has been studied in relation to various neuropsychiatric disorders [58]. However, in silico predictions suggest the missense change here will have a benign impact. No de novo exonic variants were identified in the unaffected daughter (III-2).

### Genetic variants in additional individuals pinpoint *DMXL2* as a risk gene for NDD

Much larger deletions encompassing the 15q21.2 locus are associated with ID and other features [59, 60]. A comprehensive review of public databases identified a single comparable 274-kb 15q21 loss (Fig. 2), in DECIPHER [39]. The individual is a 21-year-old Belgian male with diagnoses of mild ID, ASD, and ADHD (case 4 in Table 1). Growth parameters were within normal limits (weight 71.5 kg, height 168 cm, head circumference 56.4 cm), and there were no major congenital anomalies or notable dysmorphic features. Inheritance of the CNV could not be assessed as the proband was adopted. Seven other individuals with CNVs predicted to disrupt *DMXL2* were identified in private clinical laboratory databases (cases 5–11 in Table 1), with available phenotype data supporting a primary NDD phenotype in most. Additional CNVs disrupting *DMXL2* were identified in schizophrenia probands (cases 12–13 in Table 1) in the PGC [17], and novel loss-of-function SNVs were identified in ASD probands (cases 14–17 in Table 1) in the Autism Speaks MSSNG study [1]. Similar efforts to identify variants in *GRIK5* were not successful, with only a single exonic deletion found in the MSSNG cohort (data not shown).

**Table 1:** NDD and psychiatric phenotypes in individuals heterozygous for variants disrupting *DMXL2*

Case #	Source^a^	Variant type	Variant details	Individual	Sex	Age group	Inheritance	Reported NDD phenotype(s)				
								ASD	DD/ID	ADHD	SCZ/psychosis	Other
1	This report	Multigene loss	chr15:51,670,601–51,933,000 × 1 262,400 bp (*DMXL2*, *GLDN*)	Proband (II-1)	F	Adult	Pat	+	−	+	−	+^b^
2				Daughter (III-1)	F	Child	Mat	+	+/−	−	−	+^b^
3				Father (I-1)	M	Adult	N.D.	−	−	−	−	+^b^
4	DECIPHER	Multigene loss	chr15:51,568,830–51,843,305 × 1 274,476 bp (*DMXL2*, *GLDN*, *CYP19A1*)	Proband	M	Adult	N.D.	+	+	+	−	−
5	Canadian laboratory	Intragenic loss	chr15:51,806,694–51,843,305 × 1 36,612 bp (*DMXL2*)	Proband	M	Child	N.D.	−	+	−	−	−
6	Lineagen laboratory	Intragenic gain	chr15:51,717,028–51,792,612 × 3 75,585 bp (*DMXL2*)	Proband	M	Child	N.D.	+	−	+	−	+^c^
7		Intragenic gain	chr15:51,708,028–51,874,928 × 3 166,901 bp (*DMXL2*)	Proband	M	Child	Pat	−	+	−	−	+^d^
8		Multigene loss	chr15:51,735,136–52,620,104 × 1 884,969 bp (*DMXL2* and 12 other genes)	Proband	F	Adult	N.D.	−	+	+	−	+^e^
9				Twin sister	F	Adult	N.D.	−	−	−	+	−
10		Multigene gain^f^	chr15:50,848,381–51,741,314 × 3 892,934 bp (*DMXL2*, *GLDN*, and 7 other genes)	Proband	M	Child	N.D.	−	−	−	−	−
11				Sibling^g^	F	Child	N.D.	−	−	−	−	−
12	PGC CNV data	Multigene gain^f^	chr15:50,888,568–51,748,611 × 3 860,044 bp (*DMXL2*, *GLDN*, and 7 other genes)	Proband	M	Adult	N.D.	−	−	−	+	−
13		Multigene gain^f^	chr15:50,892,945–51,748,611 × 3 855,667 bp (*DMXL2*, *GLDN*, and 7 other genes)	Proband	F	Adult	N.D.	−	−	−	+	−
14	Autism Speaks MSSNG WGS data	LoF SNV	c.9081dupT [p.N3028_I3029delinsX]	Proband	M	Child	N.D.	+	−	−	−	−
15		LoF SNV	c.4387dupC [p.Q1463fs]	Proband^h^	F	Child	Pat	+	−	−	−	−
16		LoF SNV	c.2239C>T [p.R747X]	Proband	M	Child	Mat	+	−	−	−	−
17		LoF SNV	c.1618-2A>G	Proband	M	Child	Pat	+	−	−	−	−

Reported physical phenotypes not described elsewhere include: case #5 with mildly coarse features, dental caries, pyloric stenosis, bleeding disorder, and undergrowth; case #8 with coarctation of the aorta; case #10 with short stature and growth hormone deficiency; and case #11 with short stature and short fifth metacarpal. See the “Methods” section for details. There is no mention of psychiatric phenotyping of individuals heterozygous for an in-frame deletion in *DMXL2* in the family published by Tata and colleagues [42]

*ADHD*, attention-deficit/hyperactivity disorder; *ASD*, autism spectrum disorder; *CMA*, chromosomal microarray; *CNV*, copy number variation; *DD*, developmental delay; *F*, female; *ID*, intellectual disability; *LoF*, loss of function; *M*, male; *Mat*, maternal; *N.D.*, not determined; *Pat*, paternal; *PGC*, Psychiatric Genomics Consortium; *SCZ*, schizophrenia; *SNV*, single nucleotide variant; *WGS*, whole-genome sequencing

^a^See the “Methods” section for details

^b^See the “Results” section for details

^c^Encephalopathy, speech delay, aggression/behavior issues, and vocal tics

^d^Unilateral ptosis, hypotonia, toe walking, and some sensory and behavioral issues

^e^Bipolar affective disorder, anxiety, and one episode of catatonia

^f^Breakpoint lies within genomic extent of *DMXL2*

^g^Also with inv(5)(q13.3q33.1)

^h^Also with 15q11.2-q13.3 gain

## Discussion

We employed WGS and a multifaceted strategy to characterize a CNV of uncertain clinical significance. Diverse and converging lines of evidence suggest that haploinsufficiency of *DMXL2* is a risk factor for NDDs. The available data indicate variable expressivity and possibly incomplete penetrance (or at least age-related penetrance). Families exhibiting an apparently heritable but broad phenotype of NDD symptoms, including members with and without a clinical diagnosis of ASD, can expand our knowledge of the genetically related spectrum of disease. One of the most pressing challenges in the field is to understand the typically high degree of variable neuropsychiatric expression associated with risk variants [61, 62]. The range of symptomatology observed in this family is reminiscent of what is often observed with genomic disorders and non-recurrent large CNVs, and it should further highlight the impact of rare inherited genetic variants [18, 22, 63, 64]. In some cases, there is emerging evidence for additional deleterious variants elsewhere in the genome that may act as modifiers [65, 66]. We also identified de novo and inherited sequence variants of potential relevance in this family, including a rare missense change in *GRIK5*. The latter was not as compelling of a candidate variant as the microdeletion overlapping *DMXL2* because of (i) the predicted less-severe nature of the genetic lesion, (ii) the current absence of overt functional or model organism data at the gene level, and (iii) our inability to adequately replicate the finding in the population-scale NDD cohorts. Nonetheless, it could still be a contributor to the risk for NDD in this family. A limitation of our study was that the relatively small nuclear families precluded the identification of an individual with one, but not both, of the variants.

Our findings provide a clinical impetus to now develop and test functional hypotheses regarding disease mechanism(s). The *Dmxl2* gene is best studied in the gonadotropin-releasing hormone neurons [42, 67], where low expression in mice impedes normal dendritic development [67]. However, the phenotypes observed in knockout mice are not entirely attributable to deficient *Dmxl2* in that neuronal cell population [67]. *Dmxl2*^+/−^ mice also demonstrate neuroanatomical differences in the corpus callosum [68]. At least four of ten experimentally proven protein interactors of DMXL2 [69] are encoded by established or candidate genes for NDDs: *CYFIP2* [70, 71], *DYNC1H1* [72, 73], *MATR3* [74], and *NCKAP1* [5, 75, 76]. Both CYFIP2 and NCKAP1 shape the formation of dendritic spines via the WAVE actin-remodeling complex [77, 78]. DMXL2 protein dosage insufficiency may therefore result in structural (e.g., abnormal dendritic spines morphology) and/or functional (e.g., impaired transmission) synaptic consequences in humans, as have been observed in other genetic forms of ID, ASD, and schizophrenia [79, 80].

It remains difficult in clinical practice to interpret novel inherited CNVs, which are often labeled variants of uncertain clinical significance [49]. Incomplete penetrance and epistasis are likely underappreciated. Most approaches to CNV adjudication focus on the overlapped genes and disregard the remainder of the genome [81], in part because CMA is incapable of identifying smaller and balanced genetic variants. Whereas WGS is more comprehensive in detection [1, 24–26], sometimes also revealing complicating data for clinical interpretation, as was the case with the *GRIK5* variant. Updated guidelines are required for interpreting CNVs that take into account sequence variants [81]. Specific consideration will need to be given with respect to how to determine if two or more variants are major contributors to disease within a specific individual. The risk profile from common variants with a polygenic effect may also become clinically relevant in time, even in those individuals with a monogenic diagnosis or genomic disorder [82, 83].

Variant inheritance patterns in multigenerational pedigrees can be highly informative, with the caveat that customary assumptions (e.g., de novo suggests pathogenic, and inherited from putatively unaffected parent suggests benign) are imperfect [84–86]. The availability of samples and detailed phenotype data from three generations of a family, including offspring and parents of an individual with ASD or other NDD phenotypes, is uncommon [23]. There is substantial evidence that different rare disease-causing variants can segregate within the same family [1, 3]. Our approach here of examining multiple rare penetrant variants across generations might reveal new genes for NDD. This may especially be the case for genes involved in higher functioning forms of ASD, perhaps including *DMXL2*. Our study design also allowed for the observation that the proband’s de novo variant in *ZC3H14* was not inherited by her daughter with NDD. In contrast, both the 15q21 deletion and the *GRIK5* variant did segregate with the apparent NDD phenotypes, albeit in a simple pedigree where the a priori probability was *p* = 0.125 for any single variant [87].

## Conclusions

These results support *DMXL2* as a candidate gene for a spectrum of NDDs that includes ASD. As for virtually all (even highly penetrant) risk variants, additional modifiers of expression remain to be discovered. The robust interpretation of ultra-rare variants identified by CMA or other testing requires incorporation of WGS from affected families and population-scale data. Although currently expensive for clinical diagnostics [88], in time, we expect the same utility will be found as in this research study and WGS will become a first-tier clinical genetic test for NDDs.

## Abbreviations

ADHD: Attention-deficit/hyperactivity disorder
ADOS: Autism Diagnostic Observation Schedule
ASD: Autism spectrum disorder
CMA: Chromosomal microarray
CNV: Copy number variation
DGV: Database of Genomic Variants
ID: Intellectual disability
NDD: Neurodevelopmental disorder
PGC: Psychiatric Genomics Consortium
PIQ: Performance intelligence quotient
SNV: Single nucleotide variant
VIQ: Verbal intelligence quotient
WAIS-III: Wechsler Adult Intelligence Scale—Third Edition
WGS: Whole-genome sequencing
WISC-III: Wechsler Intelligence Scale for Children—Third Edition
